# The Influence of α-Tocopherol on Serum Biochemical Markers During Experimentally Induced Pleuritis in Rats Exposed to Dioxin

**DOI:** 10.1007/s10753-017-0536-2

**Published:** 2017-03-15

**Authors:** Ireneusz Całkosiński, Kinga Gostomska-Pampuch, Jacek Majda, Anna Leśków, Maciej Janeczek, Oleg P. Melnyk, Andrzej Gamian

**Affiliations:** 1 Independent Laboratory of Neurotoxicology and Environmental Diagnostics, Wroclaw Medical University, 51-618 Wroclaw, Poland; 2 Institute of Immunology and Experimental Therapy, Polish Academy of Sciences, 53-114 Wroclaw, Poland; 3 Department of Laboratory Diagnostics, 4th Military Hospital, 50-981 Wroclaw, Poland; 4 Department of Biostructure and Animal Physiology, Faculty of Veterinary Medicine, Wroclaw University of Environmental and Life Sciences, 50-375 Wroclaw, Poland; 5 Department of Animal Anatomy, National University of Life and Environmental Sciences of Ukraine, Kiev, Ukraine; 6 Department of Medical Biochemistry, Wroclaw Medical University, 50-368 Wroclaw, Poland

**Keywords:** pleuritis, α-tocopherol, dioxin, TCDD, inflammatory reaction, serum proteins

## Abstract

Toxicity of dioxins is wide ranging. Amongst the organs, the liver is the most susceptible to damage by dioxins. Damage caused to liver cells results in promoting inflammatory processes. The aim of this work was to evaluate whether high doses of tocopherol will change the inflammatory response, monitored by biochemical indicators, by improving liver function in rats exposed to tetrachlorodibenzo-*p*-dioxin (TCDD). The study was conducted on a population of female Buffalo rats. The animals were divided into the following groups: Control Group A—representing physiological norms for the studied diagnostic indicators; Control Group B—subjects were administered a 1% ceragenin solution to induce pleuritis; Study Group 1—where rats were administered α-tocopherol acetate for 3 weeks, after which pleuritis was induced; Study Group 2—rats were administered a single dose of 2,3,7,8-tetrachlorodibenzo-*p*-dioxin (TCDD), while 3 weeks later, pleuritis was induced; and Study Group 3—rats were administered a single dose of TCDD and next, were administered α-tocopherol acetate for 3 weeks, followed by pleuritis induction. The results clearly show that administering tocopherol in the course of inflammation causes changes to the distribution and ratio of in the serum protein fractions, including acute phase proteins. The latter proteins are indicative to the improvement in liver function and linked to protein synthesis and stimulation of the antibody-mediated immunity. Moreover, in the course of inflammation caused by exposure of rats to TCDD, tocopherol significantly affected the acute phase protein concentration.

## INTRODUCTION

The liver plays an important role in the synthesis of proteins involved in inflammatory reactions. The protein metabolism in hepatocytes is significantly affected by dioxins that are slowly and gradually accumulating in the body, mainly in adipose tissue and the liver. This accumulation leads to disruption in the synthesis of plasma proteins, i.e. albumin, α_1_, α_2_, β_1_, β_2_ and γ globulin fractions and fibrinogen. Between 25 and 75% of the daily dioxin dose is deposited in the liver [1]. Administering 2,3,7,8-tetrachlorodibenzo-*p*-dioxin (TCDD) results in changes in the endoplasmic reticulum of the hepatocytes and increases coproporphyrin concentration [2]. The latter in turn affects the synthesis of acute phase proteins, whose concentration increases rapidly during inflammation [3]. When rats are injected with dioxins, protein synthesis taking place in liver cells is disrupted on the genomic level. Pro-inflammatory interleukins, such as Il-1, Il-6 and TNF (tumour necrosis factor), act as regulators in the synthesis of plasma proteins [4–6] while increase in alanine aminotransferase activity in rats treated with TCDD is a sign of liver damage [7]. Our studies show that when rats were administered 5-μg/kg body weight of TCDD for a period of 5 weeks, they displayed significant macroscopic changes in the liver and microscopic histopathological changes in the hepatocytes. The changes manifested themselves in steatosis, and laboratory tests showed increased cholesterol concentration in plasma as well as disorders in oestrogen metabolism [8, 9]. Dioxins significantly affect the structures of the connective tissue, disrupting its linearity, promoting destructive processes and disturbing spatial distribution during regeneration processes [10]. As a result, the disrupted collagen architecture in a parenchymatous organ, such as the liver, causes destructive changes in its vascularisation. Moreover, oxidative stress was shown to significantly affect the expression of the hepatic gene responsible for the synthesis of collagen [11].

Due to dioxins’ strong pro-inflammatory characteristics, such as generation of free radicals and induction of COX-2 (cyclooxygenase-2) [12], we have attempted to eliminate the pro-inflammatory multidirectional effect of dioxins by using tocopherol’s acetate derivative. The results of our previous studies showed that administering large doses of tocopherol improves many biochemical blood indicators [13, 14]. Free radicals are generated as a result of dioxin metabolism with CYP1A1 enzymes. This process leads to formation of the reactive oxygen species (ROS) in many organs [12, 15–22]. Tocopherol deficit in cases of inflammation causes significant oxidative stress, which leads to fibrogenesis [11, 23, 24]. Vitamin E is a compound with strong antioxidative properties, which protects tissues from negative effects of the ROS [15, 25, 26]. Moreover, studies by Kloser *et al*. [27] show the antagonistic effect of tocopherol on the aryl hydrocarbon receptor (AhR). It means that the compound is helping in elimination of free radicals generated as a result of TCDD metabolism by blocking the Ah receptor and subsequently disrupting of cytochrome P450, family 1, subfamily A, polypeptide (CYP1A1) synthesis, thus preventing the generation of free radicals. This sequence of the events also provides an explanation for the layout of our study where we demonstrate beneficial changes in the biochemical parameters in rats injected with TCDD and subsequently receiving large doses of tocopherol for 3 weeks [13, 25, 28]. Lauritzen *et al*. [29] showed that during bacterial pneumonia, concentrations of the tocopherol and ascorbic acid in plasma were decreased, which led in turn to increased permeability of lysosomal membranes. The results of a study by Norazlin *et al*. [30] showed that administering tocopherol slowed the secretion of pro-inflammatory cytokines, Il-1 and Il-6, into plasma in animals exposed to nicotine. Our own studies showed similar effects in animals under the effects of TCDD, which received large doses of tocopherol for 3 weeks and in which we observed significant decreases in pro-inflammatory interleukins, especially TNF [13]. Studies by Devaraj *et al*. [31] confirmed that prolonged use of tocopherol limits the release of peroxide anions and hydrogen peroxide, decreasing Il-1 concentrations. Studies by Tsukamoto *et al*. [32] showed that administering tocopherol inhibits the expression of mRNA, Il-6 and TNF.

This work is a continuation of previous studies. Here, we decided to assess the level of acute phase plasma proteins, such as haptoglobin and transferrin, while demonstrating the preventative role of tocopherol on liver damage. It is important to note that these proteins participate in securing iron release from broken-down erythrocytes during inflammation modulated by dioxins. We have undertaken to analyse the effect of a 3-week tocopherol regimen, assuming a dose of 30-mg/kg body weight, on plasma protein and selected biochemical parameters in rats which were under the effects of TCDD and in which we induced inflammation.

## MATERIAL AND METHODS

Female Buffalo rats, weighing between 130 and 150 g, aged between 9 and 11 weeks, were inbred and provided by the Department of Pathologic Anatomy of Wroclaw Medical University. The air-conditioned rooms where the animals were held were equipped with hypertension, the air was exchanged 15 times per hour, the temperature was kept at 22 °C, humidity at 55%, and the daylight cycle was 12/12 h. The rats were kept in polystyrene cages, each holding six specimens, with free access to water and food (“Murigran”).

For the purposes of the study, the animals were divided into the following groups:

- Control Group A—representing physiological norms for the studied diagnostic indicators;
- Control Group B—rats with pleuritis, induced by administering 1% ceragenin solution, volume 0.15 ml, injected into the pleural cavity at 5/6 right intercostal space;
- Study Group 1—rats were administered α-tocopherol acetate for 3 weeks, dose 30-mg/kg body weight/day, and subsequently, pleuritis was induced following the same procedure as in the Control Group B;
- Study Group 2—rats were administered a single dose of 2,3,7,8-tetrachlorodibenzo-*p*-dioxin (TCDD) diluted in dimethyl sulfoxide (DMSO), dose 5-μg/kg body weight, 3 weeks before pleuritis was induced with ceragenin;
- Study Group 3—rats were administered a single dose of TCDD, dose 5-μg/kg body weight, 3 weeks before pleuritis was induced with ceragenin; after being administered TCDD, the animals were administered α-tocopherol acetate for 3 weeks, dose 30-mg/kg body weight/day.

The study material constituted blood drawn from the abdominal aorta of the rats, which were anaesthetized with barbiturates, i.e., 30 mg/kg body weight of sodium pentobarbital. After desanguination animals were decapitated and rats’ carcases were incinerated.

The following chemical and pharmacological agents and diagnostic tests were used in the study: model TCDD solution, diluted in DMSO, concentration 1 μg/ml, acquired at the Laboratory for Trace Analysis, Institute of Chemistry and Inorganic Technology, Kraków Polytechnic; ceragenin (Sigma); α-tocopherol acetate (Hasco-Lek); and sodium pentobarbital (Biochemie GmbH).

### Study Methods

The following biochemical tests were performed on rats’ serum and plasma:

Electrophoresis—the sera were separated in buffered agarose gel at 100-V electrical field for a period of 35 min, using a Sebia–Hydrasys system, (Horiba ABX). The gel scanning was performed with a Sebia–Hydrasys densitometer at 600 nm, and protein fraction concentration was given in g/dl;

The concentration (mg/dl) of the acute phase protein markers, i.e. complements C3 and C4, haptoglobin, transferrin, as well as IgG and IgM, was determined by immunonefelometry with a Dade Behring nefelometer, using diagnostic sets provided by the manufacturer.

### Statistical Analysis

The results were subjected to statistical analysis. We calculated the total number (*n*), arithmetical means (mean), standard deviations (SD) and ranges for minimal (min) and maximal (max) values.

Having verified whether the calculated parameters have normal distribution (by comparing the variables’ histogram with a Gauss curve diagram), the means for particular indicators for the A and B Control Groups and for the Control Group B and Study Groups, in which tocopherol and TCDD were administered, were compared using Student’s *t* test with statistical significance set at the following: *p ≤ 0.05, **0.05 > *p* ≥ 0.01, and ***p ≤ 0.001. Calculations were performed using Statistica 7.01.

## RESULTS

### Serum Protein Analysis (Table 1)

**Table 1:** Electrophoresis of Serum Proteins of Rats with Induced Pleuritis Without Dioxin and After Dioxin Exposition

Group	Hour of inflammation (h)		Albumin (g/dl)	Globulin				Total protein (g/dl)	Albumin/globulin
				α_1_ (g/dl)	α_2_ (g/dl)	β (g/dl)	γ (g/dl)		
Control	0	*n*	6	6	6	6	6	6	6
		Mean	3.700	0.460	0.318	0.902	0.445	5.780	1.790
		SD	0.520	0.230	0.078	0.201	0.181	0.550	0.520
Pleuritis	24	*n*	5	5	5	5	5	5	5
		Mean	3.040	0.924	0.506	1.042	0.334	5.850	1.090
		SD	0.110	0.123	0.027	0.121	0.094	0.280	0.130
		*p*	*	**	***	NS	NS	NS	*
	72	*n*	5	5	5	5	5	5	5
		Mean	2.990	1.108	0.462	1.048	0.226	5.830	1.050
		SD	0.090	0.022	0.085	0.051	0.065	0.110	0.070
		*p*	*	***	*	NS	*	NS	*
	120	*n*	5	5	5	5	5	5	5
		Mean	3.480	0.722	0.582	0.950	0.344	6.000	1.250
		SD	0.170	0.090	0.054	0.094	0.109	0.250	0.170
		*p*	NS	*	***	NS	NS	NS	NS
Pleuritis + vit. E	24	*n*	6	6	6	6	6	6	6
		Mean	3.460	1.068	0.413	1.257	0.313	6.510	1.140
		SD	0.270	0.130	0.093	0.312	0.102	0.590	0.050
		*p*	**	NS	NS	NS	NS	*	NS
	72	n	6	6	6	6	6	6	6
		Mean	3.370	0.848	0.512	1.040	0.322	6.090	1.240
		SD	0.350	0.115	0.085	0.155	0.125	0.600	0.060
		*p*	*	***	NS	NS	NS	NS	***
	120	*n*	6	6	6	6	6	6	6
		Mean	3.380	0.512	0.302	0.842	0.638	5.670	1.500
		SD	0.090	0.068	0.069	0.051	0.127	0.270	0.220
		*p*	NS	**	***	*	**	NS	NS
Pleuritis + TCDD	24	*n*	5	5	5	5	5	5	5
		Mean	2,310	0,908	0,552	1,304	0,890	5,960	0,630
		SD	0,170	0,107	0,054	0,126	0,082	0,260	0,070
		*p*	***	NS	NS	*	***	NS	***
	72	*n*	5	5	5	5	5	5	5
		Mean	2.970	1.052	0.492	1.058	0.256	5.820	1.040
		SD	0.040	0.147	0.051	0.062	0.062	0.110	0.050
		*p*	NS	NS	NS	NS	NS	NS	NS
	120	*n*	5	5	5	5	5	5	5
		Mean	3.690	1.092	0.316	1.386	0.334	6.820	1.190
		SD	0.070	0.068	0.084	0.269	0.100	0.330	0.110
		*p*	*	***	***	**	NS	**	NS
Pleuritis + TCDD + vit. E	24	*n*	6	6	6	6	6	6	6
		Mean	3.100	1.185	0.587	1.345	0.800	7.020	0.800
		SD	0.190	0.333	0.071	0.183	0.177	0.550	0.100
		*p*	***	NS	NS	NS	NS	**	*
	72	*n*	5	5	5	5	5	5	5
		Mean	3.820	0.474	0.400	1.254	1.188	7.090	1.140
		SD	0.160	0.231	0.122	0.129	0.153	0.360	0.050
		*p*	***	***	NS	*	***	***	**
	120	*n*	6	6	6	6	6	6	6
		Mean	3.120	0.822	0.483	0.970	0.305	5.840	1.210
		SD	0.290	0.131	0.116	0.170	0.120	0.250	0.030
		*p*	**	**	*	*	NS	***	NS

*n* total number, *SD* standard deviations

*p* statistical significance: *NS* not significant; *p ≤ 0.05; **0.05 > *p* ≥ 0.01; ***p ≤ 0.001

#### Albumins

**Fig. 1:**
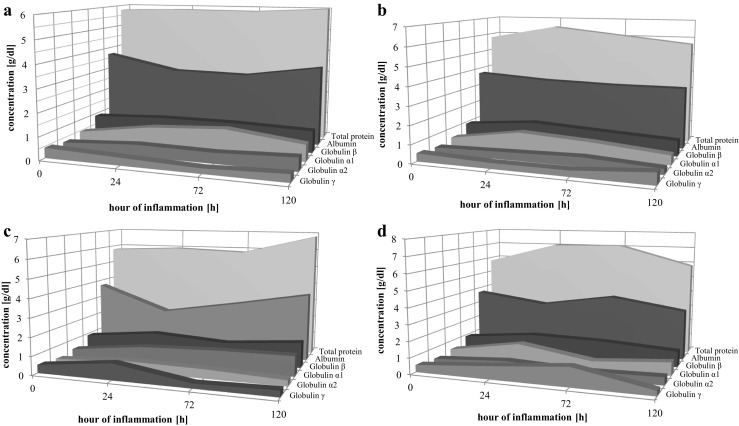
Electrophoretic separation of rat sera: **a** Control Group B; induced pleuritis; **b** Study Group 1; induced pleuritis and administration of α-tocopherol; **c** Study Group 2; induced pleuritis after administration of dioxin; **d** Study Group 3; induced pleuritis after administration of dioxins and injection of α-tocopherol.

In the Control Group B, blood samples withdrawn at 24 and 72 h since induction of the inflammation showed a significant decrease in absolute and relative albumin concentration. In the Study Group 1 with induced inflammation and administered tocopherol, we observed a significant increase in absolute and relative albumin concentration for the same time period, in comparison with the results from the Control Group B. In the Study Group 2 with induced inflammation and treated with dioxin, we observed a significant decrease in albumin concentration in comparison with that in the Control Group B. In a similar group with induced inflammation and treated with both dioxin and vitamin E, we observed an increase in the absolute and relative albumin concentration in comparison with that in the group that did not receive tocopherol (Fig. 1).

#### Globulin α_1_

In the Control Group B, with induced inflammation, the measurements were performed in three time periods and showed a significant increase in the absolute and relative concentration of this protein fraction in comparison with that in the group that had no induced inflammation. Administering tocopherol in the Study Group 1 with induced inflammation resulted in a decrease of both absolute and relative concentration of globulin α_1_, in comparison with that in the Control Group with induced inflammation. In the Study Group 2 with induced inflammation and treated with dioxin, we observed a significant increase in the concentration of this globulin fraction in the 120th hour sample in comparison with that in the Control Group B. The administration of tocopherol in a similar group that received dioxin resulted in a significant decrease in the concentration of this protein fraction in comparison with that in the group where rats were treated with dioxin that had not received tocopherol (Fig. 1).

#### Globulin α_2_

In the Control Group B, with induced inflammation, the measurements performed in three time periods showed a significant increase in the absolute and relative concentration of this protein fraction. In the Study Group that apart from induced inflammation received tocopherol, after 120 h, we observed a significant decrease in the concentration of this globulin fraction in comparison with that in the Control Group B. In the Study Group with induced inflammation and treated with dioxin, we have also observed a significant decrease in the concentration of this globulin fraction after 120 h (in comparison with that in the Control Group B). However, in the group treated with both dioxin and tocopherol, we observed a decrease in the concentration of the globulin α_2_ after 72 and 120 h since induction of the inflammation (Fig. 1).

#### Globulin β

In the Control Group B, with induced inflammation, we did not document any significant differences for this protein fraction in comparison with the results obtained for the Control Group A, without induced inflammation. The administration of tocopherol in the Study Group 1 with induced inflammation did not result in statistically significant changes, as compared to that in the previous group. After 72 and 120 h in the Study Group 2 with induced inflammation and treated with dioxin, we observed an increase in the absolute and relative concentration of the globulin β, while in the group treated with both dioxin and tocopherol, there was a significant decrease in the absolute concentration of this globulin fraction (Fig. 1).

#### Globulin γ

In the Control Group B, we observed a slight decrease of the *γ* globulin fraction in comparison with that in the Control Group A, without induced inflammation. In the Study Group 1 rats with induced inflammation and injected with tocopherol, after 120 h, we observed a significant increase in the concentration of this protein in comparison to that in corresponding sample for the Control Group B. However, γ globulin concentration after 24 h was significantly increased in the Study Group 2 in comparison with that in the animals from the Control Group B. The administration of tocopherol in the Study Group 3 with induced inflammation and treated with dioxin resulted in a significant increase of the γ globulin fraction after 24 and 72 h, in comparison with that in the group treated with dioxin, but had not received tocopherol (Fig. 1).

#### Total Serum Protein

In the Control Group B, with induced inflammation, the assays performed for samples taken at three time periods showed no differences in comparison with results obtained for the Control Group A. In the Study Group 1 after 24 h, we observed a slight increase in total serum proteins, with a tendency to remain at a constant level. In the Study Group 2, we did not observe any significant differences in the concentration of the total serum proteins after 24 and 72 h in comparison with that in the Control Group B. The total serum proteins increased in the Study Group 3 with induced inflammation and where rats were injected with both dioxin and tocopherol in comparison with those in the Study Group 2 (Fig. 1).

#### Serum Albumin to Globulin Ratio

**Fig. 2:**
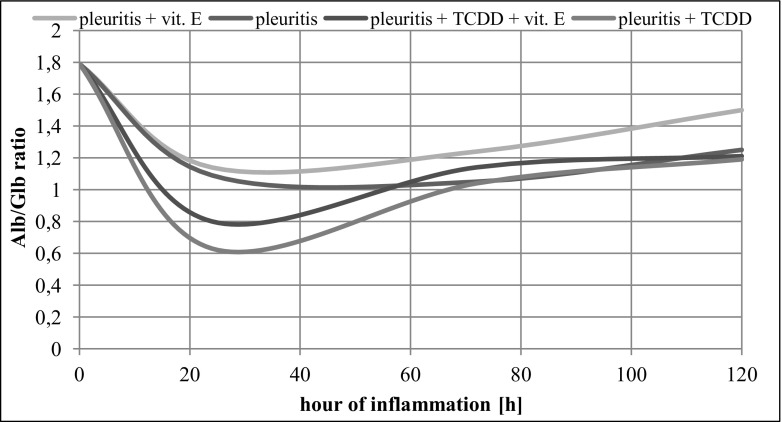
Albumin to globulin (RSA/RSG) ratio in different study groups.

Serum albumin/globulin ratio (RSA/RSG) was determined for the Control Group B (with induced inflammation) and compared to that of the Control Group A (without induced inflammation). The administration of tocopherol to rats with induced inflammation caused a slight increase of RSA/RSG in comparison with that in the Control Group B. In the Study Group with induced inflammation and treated with dioxin, RSA/RSG was slightly lower than that in the Control Group B. The injection of tocopherol to rats with induced inflammation and treated with dioxin after 24 and 72 h caused a significant increase of this indicator in comparison with rats that have not received tocopherol (Fig. 2).

### Analysis of Biochemical Indicators (Table 2)

**Table 2:** The Changes of Biochemical Inflammatory Indices With and Without Exposure to Dioxin

Group	Hour of inflammation (h)		IgG (mg/dl)	IgM (mg/dl)	C3 (mg/dl)	C4 (mg/dl)	TRF (mg/dl)	Hapt (mg/dl)
Control	0	*n*	7	7	7	7	7	7
		Mean	306.57	38.80	1.51	9.42	112.60	4.41
		SD	33.65	5.12	1.10	1.94	8.23	1.54
Pleuritis	24	*n*	7	7	7	7	7	7
		Mean	331.00	26.87	53.16	4.09	142.91	72.53
		SD	33.82	5.23	11.53	0.97	23.31	15.38
		*p*	NS	**	***	***	**	***
	72	*n*	7	7	7	7	7	7
		Mean	273.86	65.81	43.47	5.23	136.47	81.16
		SD	40.60	13.26	2.36	2.11	11.30	4.32
		*p*	NS	***	***	**	***	***
	120	*n*	7	7	7	7	7	7
		Mean	568.57	61.50	35.39	10.18	32.26	61.69
		SD	34.98	6.16	2.79	1.29	3.46	5.19
		*p*	NS	***	***	NS	***	***
Pleuritis + vit. E	24	*n*	7	7	7	7	7	7
		Mean	200.43	30.86	52.44	14.68	151.94	70.74
		SD	23.92	6.28	2.81	6.62	51.57	6.09
		*p*	***	NS	NS	***	NS	NS
	72	*n*	7	7	7	7	7	7
		Mean	181.57	82.71	54.77	14.03	142.73	74.54
		SD	15.54	9.64	4.46	2.12	27.15	5.27
		*p*	***	*	*	***	NS	NS
	120	*n*	7	7	7	7	7	7
		Mean	232.00	71.57	52.63	37.29	194.33	64.10
		SD	18.69	7.41	3.27	17.29	47.90	16.85
		*p*	***	*	***	**	***	NS
Pleuritis + TCDD	24	*n*	6	6	7	7	7	7
		Mean	168.33	19.07	50.06	13.18	189.97	79.50
		SD	26.00	3.70	1.70	2.71	44.67	9.72
		*p*	***	*	NS	***	**	NS
	72	*n*	6	6	7	7	7	7
		Mean	180.17	38.50	55.80	25.08	188.77	89.09
		SD	10.34	8.34	2.69	1.32	29.72	21.16
		*p*	***	**	*	***	***	NS
	120	*n*	6	6	7	7	7	7
		Mean	84.00	50.27	51.43	34.27	230.04	157.80
		SD	12.85	4.79	3.36	5.26	11.30	17.48
		*p*	***	**	***	***	***	***
Pleuritis + TCDD + vit. E	24	*n*	6	6	7	7	7	7
		Mean	203.00	36.43	49.97	15.96	228.87	100.23
		SD	40.98	6.63	4.60	3.75	45.06	12.27
		*p*	NS	***	NS	NS	NS	**
	72	*n*	6	6	7	7	7	7
		Mean	194.67	48.97	54.51	19.66	150.96	176.14
		SD	8.64	5.96	4.87	9.97	22.05	15.47
		*p*	*	*	NS	NS	*	***
	120	*n*	6	6	7	7	7	7
		Mean	209.00	53.15	51.70	24.17	151.11	166.79
		SD	26.97	11.91	3.74	8.14	17.64	30.01
		*p*	***	NS	NS	NS	***	NS

*IgG* immunoglobulin G, *IgM* immunoglobulin M, *C3* complement component 3, *C4* complement component 4, *TRF* transferrin, *Hapt* haptoglobin, *n* total number, *SD* standard deviations

*p* statistical significance: *NS* not significant; *p ≤ 0.05; **0.05 > *p* ≥ 0.01; ***p ≤ 0.001

#### Immunoglobulin M

**Fig. 3:**
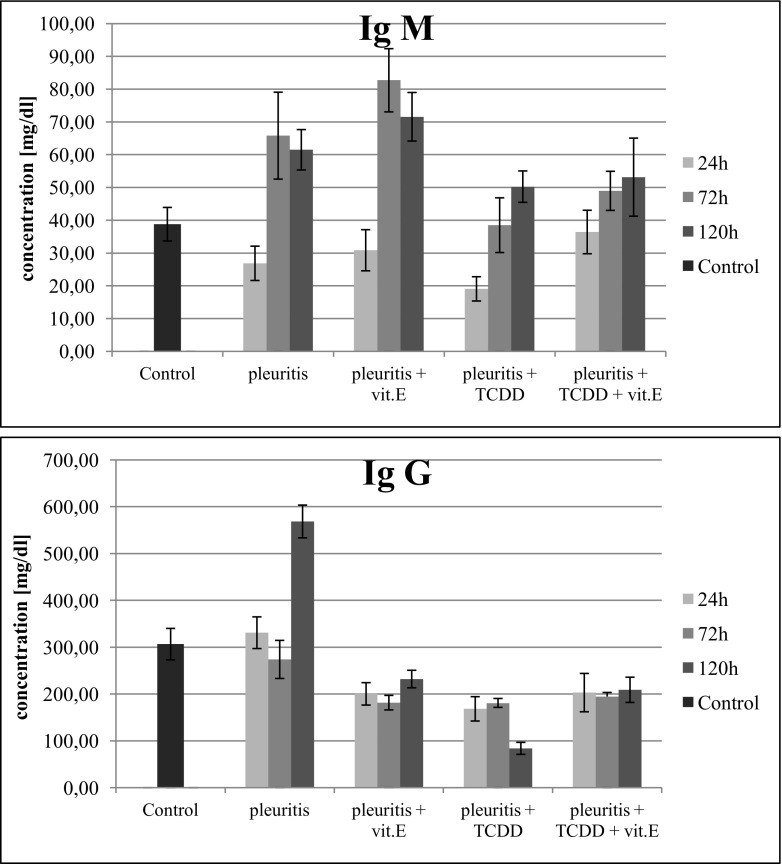
Influence of α-tocopherol on immunoglobulin M (IgM) and immunoglobulin G (IgG) concentration in serum of rats with pleuritis, with and without exposure to dioxin.

In the Control Group B, with induced inflammation, after 24 h, we observed a decrease of the immunoglobulin M (IgM) concentration, while after 72 and 120 h, we observed a significant increase in concentration of this immunoglobulin. The administration of tocopherol to rats with induced inflammation caused an increase of the IgM, while a significant decrease of this parameter was observed in rats treated with dioxin. The administration of tocopherol in the group with induced inflammation and treated with dioxin caused an increase in the concentration of the IgM after 24 and 72 h from induction of the inflammation, in comparison to that in the rats that have not received tocopherol (Fig. 3).

#### Immunoglobulin G

After 24 and 72 h, the rats in the Control Group B and Control Group A showed no significant changes in the concentration of this immunoglobulin. On the other hand, administration of tocopherol to the group of rats with induced inflammation caused a significant decrease in immunoglobulin G (IgG) concentration. When rats with induced inflammation were treated with dioxin, a significant decrease of the IgG concentration in serum was observed. Injection of both tocopherol and dioxin to rats with induced inflammation caused an increase in the concentration of this immunoglobulin in comparison to that in the rats that were not injected with tocopherol (Fig. 3).

#### Complement Component 3

**Fig. 4:**
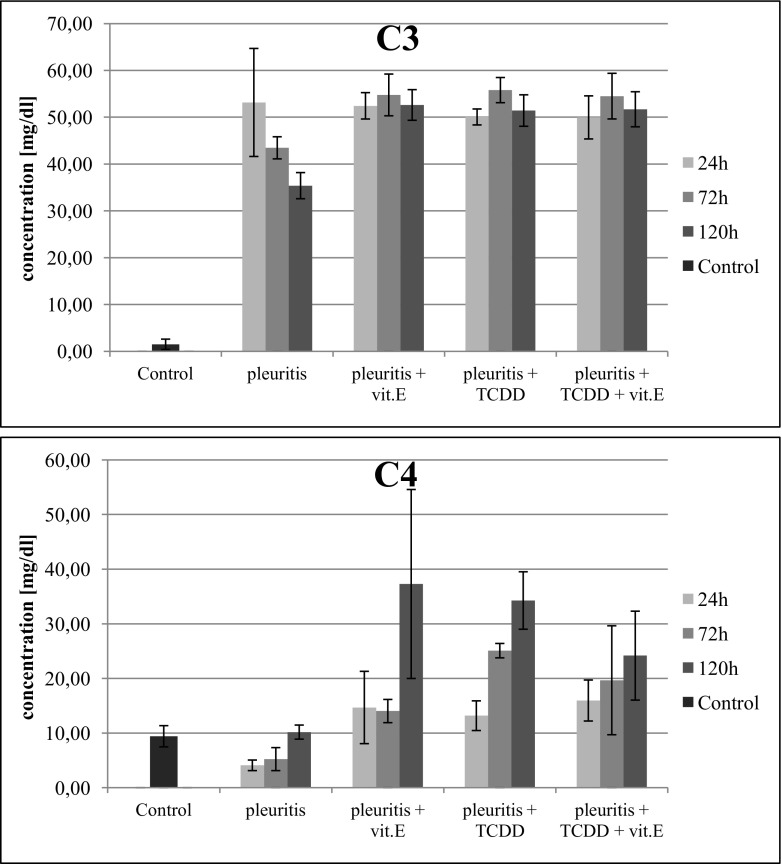
Influence of α-tocopherol on changes in the concentrations of complement C3 and C4 in serum of rats with pleuritis, with and without exposure to dioxin.

In the Control Group B, with induced inflammation, we observed 30-fold increase in the concentration of this protein, with the maximum value documented after 24 h from induction of the inflammation. In the group with induced inflammation and treated with dioxin, we observed an increase in concentration of this protein, which remained at that level throughout the experiment points. The administration of tocopherol in the group with induced inflammation and treated with dioxin did not cause any significant change in complement component 3 (C3) concentration (Fig. 4).

#### Complement Component 4

In the rats with induced inflammation (Control Group B), the concentration of component 4 (C4) decreased by 50% after 24 and 72 h, in comparison to that in the rats that were not subjected to induced inflammation (Control Group A). When rats with induced inflammation received tocopherol, after 120 h, there was fourfold increase in the concentration of this indicator (Fig. 4). In the group with induced inflammation and treated with dioxin, we also observed a significant increase in the C4 concentration. The administration of tocopherol to rats with induced inflammation and treated with dioxin did not significantly change the concentration of the C4 in all three time points (Fig. 4).

#### Transferrin

**Fig. 5:**
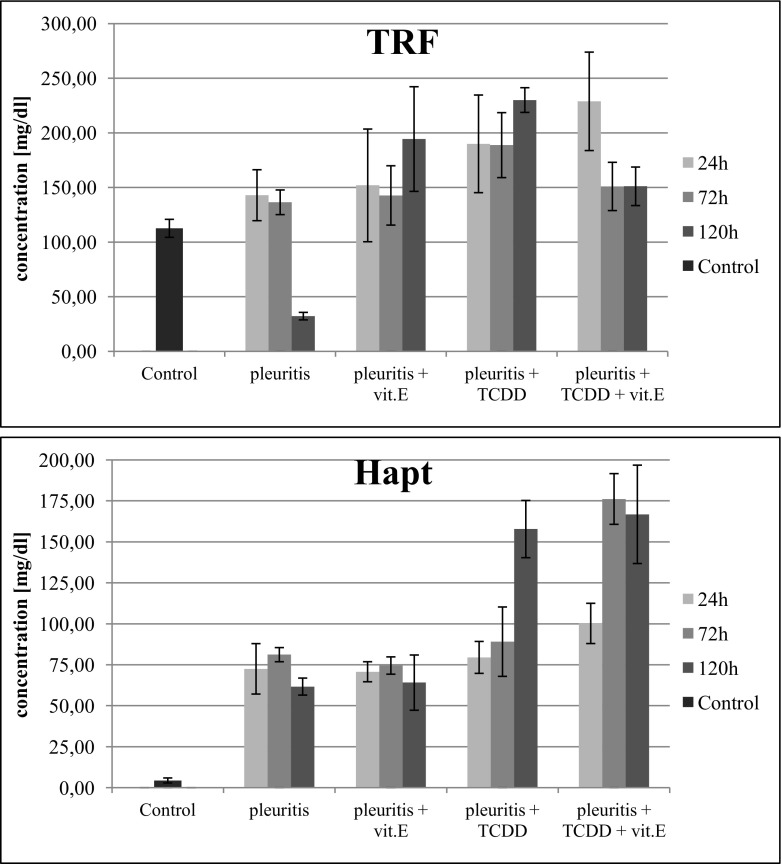
Concentration of transferrin (TRF) and haptoglobin (Hapt) in serum of rats with induced inflammation and exposed, or not to dioxin.

In the Control Group B, with induced inflammation, after 24 and 72 h, we observed a slight increase in transferrin (TRF) concentration, while after 120 h of inflammation, there was a significant decrease in the concentration of this indicator. The administration of tocopherol to rats with induced inflammation brought transferrin concentration at 120-h samples to the same level as observed after 24 and 72 h. In the group with induced inflammation and treated with dioxin, we observed a significant increase in transferrin concentration in all three time points. The administration of the tocopherol to rats with induced inflammation and treated with dioxin caused a noticeable decrease in this indicator to a level similar to that observed for rats that had induced inflammation and were injected with tocopherol (Fig. 5).

#### Haptoglobin

In the Control Group B, with induced inflammation, concentration of haptoglobin increased 16-fold. Administration of tocopherol to rats with induced inflammation did not cause significant changes to this indicator. In the group with induced inflammation and treated with dioxin, we observed an increase in the concentration of haptoglobin (Hapt), in comparison with that in the Control Group B. The administration of tocopherol to dioxin-treated rats caused an increase in Hapt, especially after 72 h (Fig. 5).

## DISCUSSION

The transferrin and albumin tested in this study are the elements of the acute phase protein group which react negatively [33–36]. The results from the group of rats subjected to induced inflammation and to tocopherol showed a lower decrease in albumin concentration after 24 and 72 h than one reported in our previous study [28]. The group of rats with induced inflammation and treated with both TCDD and tocopherol showed a smaller decrease of the albumin concentration in comparison with the analogous group that did not receive tocopherol.

The electrophoretic analysis of proteins in serum of the animals with induced inflammation and treated with tocopherol showed a decrease of α_1_ and α_2_ globulin fractions after 72 and 120 h, while these proteins were increasing in the control rats with pleuritis. On the other hand, the concentrations of γ globulin, total protein and albumin to globulin ratio (RSA/RSG) in tocopherol-treated rats displayed a tendency to increase in comparison with those in the animals that did not receive tocopherol. In overall, results suggest that administration of tocopherol to both groups of rats, with induced inflammation and treated with TCDD, and the animals that were not subjected to dioxin, led to a decrease in concentrations of α_1_, α_2_ and β globulin fractions, as well as an increase of γ globulin, total protein and the RSA/RSG. The dynamics of the changes in different protein fraction concentrations are also time-dependent, as what can be seen by the example of the α_1_ globulin fraction reaching maximum concentration after 72 h since the induction of inflammation, i.e. at the same time as the other proteins such as albumin, γ globulin and the albumin to globulin ratio. Moreover, in this time period, we observed the maximum increase in leucocyte count in peripheral blood, together with a decrease in neutrophils and fall in both erythrocyte count and haematocrit, the latter being linked to albumin to globulin ratio [25, 37]. Vos *et al*. [38] showed that dioxins cause an increase in α and β globulins, and this observation is supported by results presented in this study. The aforementioned study used a similar experimental model, but not as extensive as the one presented in this study. Here, we analysed the changes in composition of the serum proteins in rats with induced inflammation and subjected to the TCDD treatment. In animals that received tocopherol, acute phase proteins’ concentration changed between the 72nd and 120th hour of inflammation. The results did not show similar time period-related interdependencies regarding biochemical parameters, as in the case of the previously described control group with induced inflammation, where, depending on the indicator, the peak of changes occurred between the 72nd and 96th hour of inflammation [28]. In a light of these observations, we hypothesize that rats in the groups that received tocopherol, the monitored indicators do not reach their potential maximal values, but rather remained at a constant level. This suggestion is supported by the results obtained for other biochemical parameters monitored when using a similar experimental model [13].

The observed increase in IgM concentration at three experimental time periods (24, 72 and 120 h) in the control group with induced inflammation also occurred in the study group that received tocopherol, as what can be construed as the effect of this vitamin on the synthesis of the abovementioned immunoglobulin. We have also shown a decrease in IgG concentration in the study group with induced inflammation and administered tocopherol.

The immunosuppressive effect of dioxins [39, 40] has also been confirmed in this study through the analysis of IgG and IgM in the study group of rats with induced inflammation and treated with TCDD. In this case, IgG concentration decreased sixfold and IgM concentration twofold in comparison with those in the control group with induced inflammation. Suppression in the production of several classes of immunoglobulins, i.e. IgG, IgE and IgM in B lymphocyte cultures, was shown by Karras *et al*. [41].

When one study group received tocopherol for 3 weeks, from the moment when dioxin was administered and until the inflammation was induced, we observed an increase in IgG and IgM in comparison with those in the control group that had not been treated with tocopherol. This shows that synthesis of these two immunoglobulins can improve after administering tocopherol. As inflammation promotes the oxidative stress [29], it causes a release of large amounts of free radicals [42–45]. The oxidative stress is strengthened after administration of dioxins.

In spite of the fact that transferrin is recognized as one of the acute phase proteins, which are known to decrease during inflammation, in this study, this protein’s concentration remained unchanged in the early stages of the inflammation. Only after 72 h into the experiment, there was a significant decrease in transferrin concentration. In the studies conducted on monkeys that received small doses of TCDD, Riecke *et al*. [46] demonstrated an increase in transferrin concentration in the liver. The analysis of our results indicates that administration of the tocopherol to rats with induced inflammation results in a reverse type of response, manifested by a considerable increase in TRF concentration. We have also observed that rats with inflammation exposed to dioxins had significantly increased transferrin concentration. The administration of tocopherol in a group of rats treated earlier with dioxin decreased the TRF concentration in comparison to that in the animals that have not received the vitamin E. Transferrin is known for its role in capturing iron from heme, released from broken-down erythrocytes, and transporting it to the endothelial reticulum. This process is probably associated with the significant decrease in the erythrocyte and haemoglobin count in later stages of inflammation [47]. Pro-inflammatory factors, such as IL-1, IL-6 and TNF, stimulate hepatocytes to synthesize acute phase proteins, haptoglobin and transferrin, which participate in preventing iron complexes and its free form from becoming available to bacteria [48, 49]. Notably, in rats treated with dioxin, during inflammation, transferrin concentration increased while the erythrocyte and haemoglobin count decreased.

The analysis of serum haptoglobin concentration in the group of rats with induced inflammation and treated with tocopherol showed no significant differences in comparison with that in a similar group which have not received the vitamin E. In the group of animals exposed to dioxin, a significant increase in haptoglobin concentration, together with an increase in transferrin concentration, was found in the 120-h sample. Administering tocopherol in the group with induced inflammation and treated with dioxin was associated with a significant increase in haptoglobin concentration, the phenomenon that can be attributed to the improvement in the synthesis of this protein. However, production of the haptoglobin might be stimulated by decrease in serum pro-inflammatory interleukins IL-1β, IL-6 and TNF, that is caused by administration of tocopherol, as described by Ahmad *et al*. [23], Jialal *et al*. [50], Norazlin *et al*. [30] and Wang *et al*. [51].

Analysis of complement C3 and C4 concentrations shows that significant differences between C3 and C4 levels appear only in the control group with induced inflammation. In the initial stage of the inflammation, complement C4 concentration has significantly decreased, whereas complement C3 concentration has increased, even though C3 is characterized as a low-response protein according to Koj’s classification [36]. Tocopherol administered to the group with induced inflammation exerts its effect by causing an increase in both complement C3 and C4 concentrations. The complement C3 and C4 concentrations have increased in serum of rats with induced inflammation and treated with TCDD. When rats with induced inflammation and treated with dioxin received tocopherol, there was no significant change in the concentration of these proteins. The elevated complement C3 and C4 concentrations in rats with inflammation and treated with dioxin may cause a significant decrease of the erythrocyte count, due to haemolytic properties of these proteins, or also due to morphological disorders in erythrocyte membranes occurring as a result of increased oxidative stress caused by the effects of TCDD [47].

## CONCLUSIONS

In cases of inflammation, tocopherol contributes to significant changes in serum and plasma protein concentrations. By using electrophoretic separation of the serum proteins, we have ascertained that tocopherol stimulates an increase in albumin and γ globulin concentration as well as the albumin to globulin ratio, whereas it prevents the increase of α_1_ and α_2_ globulin concentrations. Moreover, the increase of complement C4 concentration intensifies, while the period over which increased transferrin and complement C3 concentrations are being maintained has significantly lengthened without any significant effect on haptoglobin concentration. In cases of inflammation, tocopherol intensifies the increase of immunoglobulin M concentration and lowers immunoglobulin G concentration. The presented results clearly show that in case of inflammation, administration of tocopherol leads to changes in the serum protein fractions, including acute phase proteins, as what argues in favour of improved liver function associated with protein synthesis and stimulation of the antibody-mediated immunity.

Tocopherol significantly affects acute phase protein concentration in inflammation after administration of TCDD by limiting the dioxin-induced increase in transferrin concentration in advanced stages of this process. However, it causes a significant increase in concentrations of haptoglobin, and it does not accelerate the appearance of complement C3 and C4 proteins. This proves that the effect of TCDD on some processes involved in inflammatory response is so long-lasting that even extended use of tocopherol cannot affect the course of these processes.
